# Exploring healthcare professionals’ knowledge, attitude, and practices towards pharmacovigilance: a cross-sectional survey

**DOI:** 10.1186/s40545-020-00287-3

**Published:** 2021-01-04

**Authors:** Rabia Hussain, Mohamed Azmi Hassali, Furqan Hashmi, Tayyaba Akram

**Affiliations:** 1 Commonwealth Pharmacists Association, London, E1W 1AW UK; 2 Faculty of Pharmacy, The University of Lahore, Lahore, 54590 Pakistan; 3 School of Pharmaceutical Sciences, Universiti Sains Malaysia, 11800 Pulau Pinang, Malaysia; 4 University College of Pharmacy, University of the Punjab, Lahore, 54000 Pakistan; 5 School of Mathematical Sciences, Universiti Sains Malaysia, 11800 Pulau Pinang, Malaysia

**Keywords:** Pharmacovigilance, Adverse drug reaction, Medicines safety, Healthcare professionals, Drug regulatory authority of Pakistan, Adverse drug reaction reporting

## Abstract

**Background:**

Spontaneous reporting of adverse drug reactions (ADRs) is a method of monitoring the safety of drugs and is the basic strategy for the post-marketing surveillance of the suspected drugs. Despite its importance, there is very little reporting of ADRs by healthcare professionals. The present study has evaluated the knowledge, attitude and practices of health care professionals (HCPs) regarding pharmacovigilance activities in Lahore, Pakistan.

**Methods:**

A cross-sectional questionnaire-based survey was employed, and a convenience sampling was opted to collect the data among physicians, pharmacists and nurses working in tertiary care public hospitals of Lahore, Pakistan from September 2018 to January 2019.

**Results:**

Of the 384 questionnaires distributed, 346 health care professionals responded to the questionnaire (90.10% response rate). Most participants had good knowledge about ADR reporting, but pharmacist had comparatively better knowledge than other HCPs regarding ADR (89.18%) pharmacovigilance system (81.08%), its centres (72.97%) and function (91.89%). Most of the participants exhibited positive attitude regarding ADR reporting, such as 49.1% of physicians (*P* < 0.05), 70.2% pharmacists and 76.1% nurses showed a positive attitude that they are the most important HCPs to report an ADR. About 64.3% of physicians (*P* < 0.05) emphasized that consulting other colleagues is important before reporting an ADR. Of all, 77.7% physicians, 75.7% pharmacists and 68% of nurses had positive attitude that ADR reporting is a professional obligation and 67.6% of the pharmacists stated that they have reported ADRs in their workplace and 77.2% nurses have verbally reported ADRs to the concerned personnel or department.

**Conclusion:**

Among all HCPs, pharmacists had better knowledge about ADR reporting and pharmacovigilance. All HCPs had positive attitude and inclination towards ADR reporting. The discrepancies were observed in the practices related to ADR reporting, whereas most of the participants including physicians and nurses did not report any ADR. Based on the above, strategies are needed to educate, train, and empower the HCPs in the domain of pharmacovigilance.

## Introduction

The World Health Organization (WHO) defines an adverse drug reaction (ADR) as, “a response which is noxious and unintended, and which occurs at doses normally used in humans for the prophylaxis, diagnosis, or therapy of disease, or for the modification of physiological function [1]. ADRs pose a major global concern causing substantial morbidity and mortality, thus requiring a surveillance system which could monitor the effects of drugs in the wider population [2].

Spontaneous or voluntary reporting of ADRs refers to the passive reporting of ADRs by healthcare professionals (HCPs) or patients and is the basic strategy for the post-marketing surveillance of the suspected drugs [3]. These spontaneous reporting systems are established to detect ADRs efficiently and inexpensively and the success of these systems depends on the quality of reports submitted by HCPs [3, 4]. ADR reporting through voluntary submission has started in the early sixties in many western countries and the United Kingdom was the first country to start this program in 1964. This enabled physicians and pharmacists to report ADRs, which served as a tool for the identification of new or suspected ADRs [5]. Data collected from such reports can be influenced by the differences in population, medicine use, and alternative therapies, hence every country must establish a national pharmacovigilance system [6–9]. Though many countries including Nepal, Sri Lanka, Qatar, Oman, Bahrain, Jordan, Saudi Arabia, Yemen, Lebanon, and Egypt are members of the Uppsala Monitoring Centre, but the pharmacovigilance system is still in the development stage in these countries [10]. Pakistan’s National pharmacovigilance system has been recognized as the WHO member for Program for International Drug Monitoring (PIDM) in 2018 [11]. The National Pharmacovigilance Centre (NPC) is established under the Drug Regulatory Authority of Pakistan (DRAP) [12]. With reference to the establishment of the NPC in the country, although the DRAP has launched an online reporting form for all HCPs nationwide, but the reporting from HCPs is very low [13–16].

Healthcare professionals are responsible for the identification, documentation, and reporting of ADRs and their contribution is essential to the early detection and reporting of an ADR [17]. However, many factors including lack of knowledge, ambiguity about ADR and its reporting system and difficulties in understanding of reporting system influence the reporting of an ADR by a healthcare provider [18, 19]. Thus, to improve the ADR reporting, it is very important to understand the knowledge, attitude and practices of HCPs in ADR reporting as many studies have shown that, the optimization of pharmacovigilance (PV) knowledge, attitude and practice is crucial to formulate strategies for the improvement of ADR reporting system [20].

Concerning the scarcity of literature about the evaluation of pharmacovigilance activities in Pakistan, assessment of the knowledge, attitude and practices are critical to study. Thus, keeping in view the available literature, this quantitative survey has been developed to evaluate the trends of pharmacovigilance activities in the city of Lahore, Pakistan.

This survey has adopted a triangulation of qualitative and quantitative methods, such as the findings from the qualitative study were used to design the questionnaire to assess the current scenario of knowledge, attitude and practices of pharmacovigilance activities in Lahore, Pakistan [19–21]. By conducting the quantitative study, issues within the system can be identified and the extent of such issues can be evaluated.

## Methods

### Study design and population

A cross-sectional study design was selected and the sampling strategy that was considered appropriate for a cross-sectional survey was convenience sampling. In convenience sampling, participants were selected according to their convenient accessibility and proximity [22].

To calculate an appropriate representative sample from the targeted population, the Cochran formula was used and a sample size of 384 HCPs was calculated to demonstrate a statistically significant result for the study [23].

The selected participants were HCPs including physicians, pharmacists and, nurses working full time, as permanent employee in tertiary care public hospitals of Lahore. The participants were selected only, if:

1. Participants were registered with the relevant provincial/national council.
2. Participants provided a written consent to the researcher.

The study was approved by the Humans Ethics Committee (HEC), University College of Pharmacy, University of the Punjab, Lahore, Pakistan with reference no. HEC/PUCP/1943.

### Development of survey questionnaire

The survey questionnaire was developed from the literature review based on the knowledge, attitude, and practices of HCPs in relation to the adverse drug reaction reporting and the understanding of pharmacovigilance activities [18–20, 24–31].

The questionnaire was comprised of five main sections with 35 items covering the following components: The first part of the questionnaire consisted of 18 items, which were divided into three main sections including knowledge about ADR reporting, types of ADRs to be reported and, knowledge about pharmacovigilance. The response for this part of the questionnaire was provided in the form of ‘yes’, ‘no’ and ‘don’t know’. The second part was comprised of 7 items exploring the attitudes of the HCPs and the factors that cause a positive or negative change in the attitude of these HCPs. These items were provided in the form of statements and HCPs were provided with a 5-point Likert scale (1 = ‘strongly disagree’, 2 = ‘disagree’, 3 = ‘neutral’, 4 = ‘agree’ and 5 = ‘strongly agree’) to indicate their disagreement or agreement. Moreover, the response (1 = ‘strongly disagree’, 2 = ‘disagree’) was labelled as negative attitude and (4 = ‘agree’ and 5 = ‘strongly agree’) as a positive attitude. The third part of the questionnaire covered 10 items based on the practices of HCPs towards ADR reporting. It was comprised of 4 items for reporting practices and 6 items related to the modes of reporting. Whereas the response was provided in the form of ‘yes’, and ‘no’.

### Validation of survey questionnaire

Before the survey was implemented, the questionnaire was tested for its face and content validity. Two academicians from the School of Pharmaceutical Sciences, Universiti Sains Malaysia were asked to review the questionnaire in terms of its clarity, relevance, and ease to understand the questions. The response from these reviewers was considered before finalizing the questionnaire.

Before the implementation of the actual survey, the questionnaire was pilot tested on 30 HCPs, who were excluded from the actual study. This helped in the reliability assessment of the questionnaire by calculating Cronbach’s coefficient alpha. The internal consistency of the questionnaire was 0.72.

### Data collection

The survey-based data was collected from the HCPs working in tertiary care public hospitals of Lahore, Pakistan during September 2018 to January 2019. The self-administered questionnaire was distributed among HCPs. A statement about the research project and consent form was also distributed. It was later on collected together with the filled questionnaire from the participants. The response from the participants was entirely anonymous and voluntary.

### Statistical analysis

The data from the questionnaire was added directly to the Statistical Package for the Social Sciences (SPSS) version 24 (IBM 2013) and rechecked for any incorrect entry. To describe demographic variables, descriptive statistics were used, percentages and frequencies were used to express categorical variables. Data that emerged from domains using the Likert scale were found non-parametric. The association between the demographics of the respondents and knowledge, attitude, practices were calculated using the Kruskal Wallis test, whereas the *p* value < 0.05 was considered as significant [32].

## Results

### Demographics data

A total of 384 (*n* = 384) questionnaires were distributed to HCPs (based on inclusion criteria). The returned questionnaires were collected by the researcher with a response rate of 90.10% (*n* = 346) for all participating HCPs. The majority of the HCPs who contributed for this survey were nurses, 56.9% (*n* = 197), followed by physicians, 32.4% (*n* = 112) and pharmacists, 10.7% (*n* = 37). Most participating HCPs were females, i.e., 82.9% (*n* = 287), while only 17.1% (*n* = 59) of males responded to the survey. About 56.9% (*n* = 197) of participants belong to the age group of 31–35, while only 1.2% (*n* = 4) were from age above 45 with an overall mean age of 32.30 years. About 70.8% (*n* = 245) participants were graduates and only 19.7% (*n* = 68) had specialization in their field. Majority of participating HCPs, i.e., 51.4% (*n* = 178), had a job experience of 5 years, while 3.2% (n = 11) had an experience of more than 20 years, with an overall mean experience of 4.31 years. The demographics of the participants are given in Table 1.

**Table 1:** Demographics of the participants (*n* = 346)

Category	Subcategory	Frequency (percentage) *n* (%)
Healthcare professional type	Physicians	112 (32.4)
	Pharmacists	37 (10.7)
	Nurses	197 (56.9)
Gender	Male	59 (17.1)
	Female	287 (82.9)
Age (years)	25–30	57 (16.5)
	31–35	197 (56.9)
	36–40	72 (20.8)
	41–45	16 (4.6)
	45 or above	4 (1.2)
Mean and SD	32.30 ± 4.69	
Qualification	Bachelors	245 (70.8)
	Masters	33 (9.5)
	Specialization	68 (19.7)
Experience	5 years or below	178 (51.4)
	6–10 years	118 (34.1)
	11–15 years	26 (7.5)
	16–20 years	13 (3.8)
	20 or above	11 (3.2)
Mean and SD	4.31 ± 2.56	

### Knowledge about adverse drug reaction reporting

**Fig. 1:**
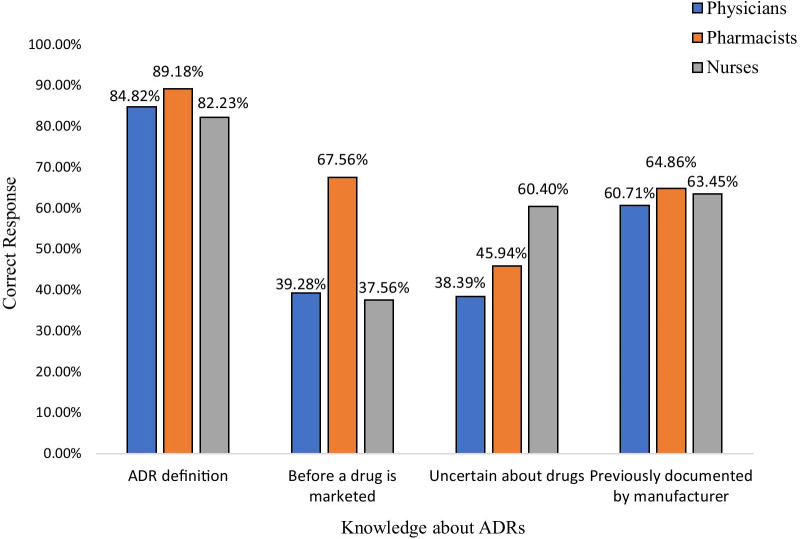
Knowledge of HCPs about adverse drug reactions

The current study intended to assess the knowledge of the HCPs towards adverse drug reaction reporting. The majority of physicians, 84.82% (*n* = 95), pharmacists, 89.18% (*n* = 33) and nurses, 82.23% (*n* = 162) gave correct response of basic definition of ADR. Most of the physicians and nurses gave incorrect responses regarding whether if the serious ADRs are known before the marketing of a new drug. While most pharmacists responded correctly, and the association was found significant with a *p* value < 0.005. About 60.40% nurses, (*n* = 119) gave correct answer that ADRs should be reported only about the suspected drugs, while 57.14% physicians (*n* = 64) and 48.64% pharmacists (*n* = 18) responded otherwise, and a negative association was found (*p* < 0.005) with physicians to the response (Table 2). Majority physicians, 60.71% (*n* = 68), 64.86% pharmacists (*n* = 24) and 63.45% nurses (*n* = 125) responded incorrectly, that previously reported ADRs by manufacturers should not be reported (Fig. 1).

**Table 2:** Knowledge of HCPs about adverse drug reaction reporting

Knowledge about ADRs	Category	Correct	Incorrect	Don’t know	*p* value
		Frequency (percentage)	Frequency (percentage)	Frequency (percentage)	
		*n* (%)	*n* (%)	*n* (%)	
ADRs can be recognized as noxious, unwanted effects of drugs	Physicians	95 (84.82)	8 (7.14)	9 (8.03)	0.534
	Pharmacists	33 (89.18)	3 (8.10)	1 (2.70)	
	Nurses	162 (82.23)	21 (10.65)	13 (6.59)	
All serious ADRs are known before a drug is marketed	Physicians	44 (39.28)	63 (56.25)	5 (4.46)	0.013
	Pharmacists	25 (67.56)	10 (27.02)	2 (5.40)	
	Nurses	74 (37.56)	108 (54.82)	15 (7.62)	
ADRs should not be reported if uncertain about the medicine that caused the adverse effect	Physicians	43 (38.39)	64 (57.14)	5 (4.46)	0.000
	Pharmacists	17 (45.94)	18 (48.64)	2 (5.40)	
	Nurses	119 (60.40)	76 (38.57)	2 (1.01)	
ADRs which are previously documented by manufacturers, need not to be reported again	Physicians	68 (60.71)	31 (27.67)	13 (11.60)	0.639
	Pharmacists	24 (64.86)	8 (21.62)	5 (13.51)	
	Nurses	125 (63.45)	54 (48.21)	18 (16.07)

### Knowledge about types of ADRs to be reported

**Fig. 2:**
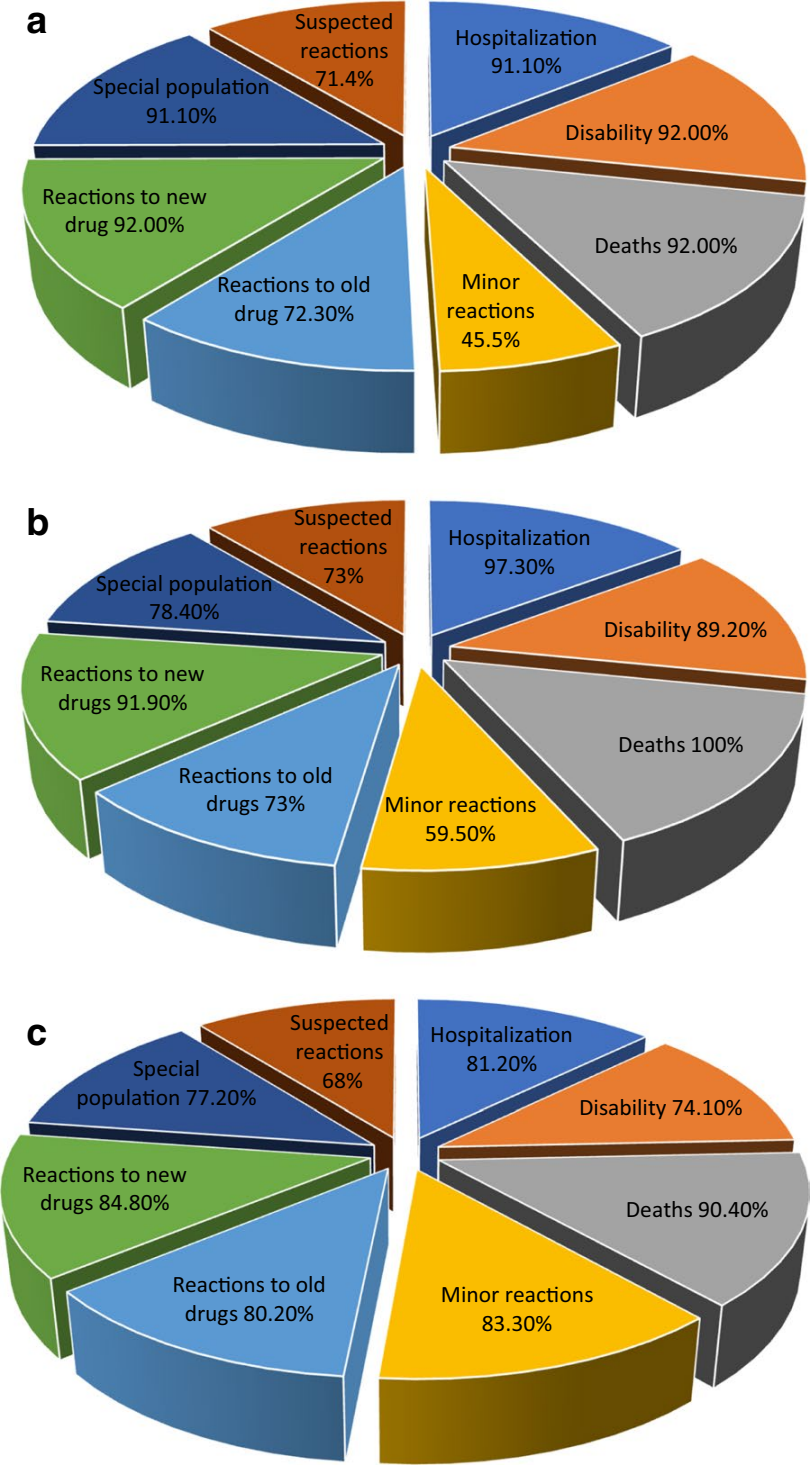
**a** Types of ADRs to be reported (by physicians). **b** Types of ADRs to be reported (by pharmacists). **c** Types of ADRs to be reported (by nurses)

A large majority of the physicians, pharmacists, and nurses were of the view that suspected reactions (suspected drug is uncertain), reaction causing hospitalization, persistent disability or death should be reported (Table 3). The majority nurses 83.3% (*n* = 165) were agreed, that minor reactions should also be reported. Most participants considered that reactions to old drugs, to newly introduced drugs in the market or reaction in the special population should also be reported, see Fig. 2a–c.

**Table 3:** Types of ADRs to be reported

ADRs to be reported	Physicians	Pharmacists	Nurses
	Frequency (percentage) *n* (%)	Frequency (percentage) *n* (%)	Frequency (percentage) *n* (%)
Suspected reactions (suspected drug is uncertain)	81 (71.4)	27 (73.0)	134 (68.0)
Reaction causing hospitalization	102 (91.1)	36 (97.3)	160 (81.2)
Reaction causing persistent disability	103 (92.0)	33 (89.2)	146 (74.1)
Reaction causing death of the patient	103 (92.0)	37 (100)	178 (90.4)
Minor reactions such as vomiting and diarrhea	51 (45.5)	22 (59.5)	165 (83.3)
Reactions to old drugs	81 (72.3)	27 (73.0)	158 (80.2)
Reactions to newly introduced drugs in the market	103 (92.0)	34 (91.9)	167 (84.8)
Any reaction in special population, e.g., children	102 (91.1)	29 (78.4)	152 (77.2)

### Knowledge about pharmacovigilance

**Fig. 3:**
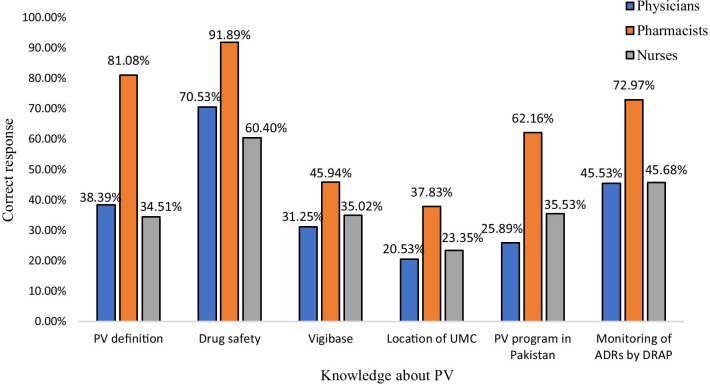
Knowledge of HCPs about pharmacovigilance

When participants were asked about the definition of pharmacovigilance, among all, majority of the pharmacists 81.08% (*n* = 30) responded correctly to the definition. The majority of physician 70.53% (*n* = 79), pharmacists 91.89% (*n* = 34), and nurses 60.40% (*n* = 119) responded with the right answer that pharmacovigilance ensures the safety of drugs, as given in Table 4. About half of the participants did not know about Vigibase, and only pharmacists correctly responded to the location of International Drug Monitoring Centre and the existence of a pharmacovigilance program in Pakistan, respectively. Among all participants, majority pharmacists 72.97% (*n* = 27) were aware that DRAP is responsible for the monitoring of ADRs in Pakistan, see Fig. 3.

**Table 4:** Knowledge about pharmacovigilance

Knowledge about PV	Category	Correct	Incorrect	Don’t know	*p* value
		Frequency (percentage)	Frequency (percentage)	Frequency (percentage)	
		*n* (%)	*n* (%)	*n* (%)	
Definition of pharmacovigilance	Physicians	43 (38.39)	56(50.0)	13 (11.60)	0.000
	Pharmacists	30 (81.08)	6 (16.21)	1 (2.70)	
	Nurses	68 (34.51	109 (55.32)	20 (10.51)	
I understand that the most important purpose of Pharmacovigilance is to ensure safety of drugs	Physicians	79 (70.5)	18 (16.07)	15 (13.39)	0.000
	Pharmacists	34 (91.89)	3 (8.10)	0	
	Nurses	119 (60.40)	32 (16.24)	46 (23.35)	
I am aware of "Vigibase" online Database for reporting adverse drug reaction by the World Health Organization	Physicians	35 (31.25)	52 (46.42)	25 (22.32)	0.073
	Pharmacists	17 (45.94)	18 (48.64)	2 (5.40)	
	Nurses	69 (35.02)	82 (41.62)	46 (23.35)	
The International Center for Adverse Drug Reaction Monitoring is located in Sweden	Physicians	23 (20.53)	27 (24.10)	62 (55.35)	0.009
	Pharmacists	14 (37.83)	11 (29.72)	12 (32.43)	
	Nurses	46 (23.35)	30 (15.22)	121 (61.42)	
I am aware of the existence of pharmacovigilance program in Pakistan	Physicians	29 (25.89)	49 (43.75)	34 (30.35)	0.000
	Pharmacists	23 (62.16)	10 (27.02)	4 (10.81)	
	Nurses	70 (35.53)	73 (37.05)	54 (27.41)	
In Pakistan Drug Regulatory Authority of Pakistan is responsible for monitoring of ADRs	Physicians	51 (45.53)	28 (25.00)	33 (29.46)	0.016
	Pharmacists	27 (72.97)	2 (5.40)	8 (21.62)	
	Nurses	90 (45.68)	28 (14.21)	79 (40.10)

### Attitudes related to ADR reporting

**Fig. 4:**
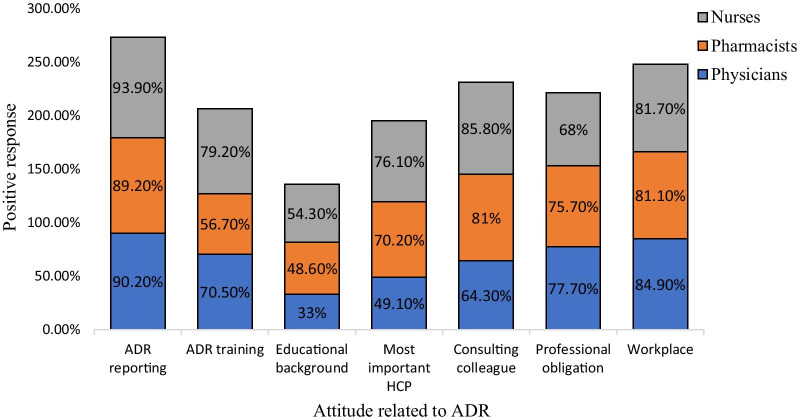
Attitude of HCPs related to ADR reporting

The majority participants considered ADR reporting as a way to improve the safety of medicines. About 35.7% physicians (*n* = 40), 56.7% pharmacists (*n* = 21, *p* < 0.05) and 41.6% nurses (*n* = 82) agreed that ADR reporting related information can be better learnt during the internship/training/clinical posting. When participants were asked, if their educational background has provided enough information about ADR reporting, 42.9% of physicians (*n* = 48, *p* < 0.05) showed disagreement over it, but 54.3% nurses (*n* = 107) and 48.6% pharmacists (*n* = 18) agreed with the statement. About 49.1% of physicians (*n* = 55, *p* < 0.05), 70.2% pharmacists (*n* = 26) and 76.1% nurses (*n* = 150) showed a positive attitude that they are the most important HCPs to report an ADR. Majority participants, i.e., 64.3% of physicians (*n* = 72, *p* < 0.05) emphasized that consulting other colleagues is important before reporting an ADR. Of all, 77.7% physicians (*n* = 87), 75.7% pharmacists (*n* = 28) and 68% of nurses (*n* = 134) had a positive attitude that ADR reporting is a professional obligation, as given in Table 5. Almost half of the participants including 49.1% physicians (*n* = 55), 51.4% pharmacists (*n* = 19) and 41.1% nurses (*n* = 81) had a positive inclination that the reporting of an ADR should be mandatory for all HCPs. Similarly, 41.1% physicians (*n* = 46) and 48.6% pharmacists (*n* = 18) and 38.1% nurses (*n* = 75) had positive attitude that ADR should be confirmed before reporting to the hospital management. A large majority of the participants, i.e., 84.9% physicians (*n* = 95), 81.1% pharmacists (*n* = 30) and 81.7% nurses (*n* = 161) positively agreed that workplace should encourage HCPs about ADR reporting, as illustrated in Fig. 4.

**Table 5:** Attitudes related to ADR reporting

Attitudes related to ADR	Category	Negative	Neutral	Positive	*p* value
		Frequency (percentage)	Frequency (percentage)	Frequency (percentage)	
		*n* (%)	*n* (%)	*n* (%)	
I believe that the adverse drug reaction ADR reporting is an important activity to improve safety of medicines	Physicians	11 (9.8)	0 (0)	101 (90.2)	0.761
	Pharmacists	4 (10.8)	0 (0)	33 (89.2)	
	Nurses	5 (2.5)	7 (3.6)	185 (93.9)	
I believe that Information on reporting ADRs are better learnt during the internship /ADR training/clinical posting	Physicians	9 (8)	24 (21.4)	79 (70.5)	0.019
	Pharmacists	4 (10.8)	12 (32.4)	21 (56.7)	
	Nurses	14 (7.1)	27 (13.7)	156 (79.2)	
I believe that my educational background has provided me with enough information about ADR reporting	Physicians	48 (42.9)	27 (24.1)	37 (33)	0.000
	Pharmacists	8 (21.6)	11 (29.7)	18 (48.6)	
	Nurses	48 (24.4)	42 (21.3)	107 (54.3)	
I believe that I am the most important health care professional to report ADRs	Physicians	27 (24.1)	30 (26.8)	55 (49.1)	0.000
	Pharmacists	4 (10.8)	7 (18.9)	26 (70.2)	
	Nurses	19 (9.7)	28 (14.2)	150 (76.1)	
I believe that consulting colleagues and other healthcare professional is important before reporting an ADR	Physicians	13 (11.6)	27 (24.1)	72 (64.3)	0.000
	Pharmacists	5 (13.5)	2 (5.4)	30 (81)	
	Nurses	9 (4.5)	19 (9.6)	169 (85.8)	
I believe that reporting an adverse drug reaction is a professional obligation	Physicians	9 (8.1)	16 (14.3)	87 (77.7)	0.065
	Pharmacists	1 (2.7)	8 (21.6)	28 (75.7)	
	Nurses	41 (20.8)	22 (11.2)	134 (68)	
I believe that workplace environment should encourage reporting an ADR	Physicians	7 (6.3)	10 (8.9)	95 (84.9)	0.347
	Pharmacists	2 (5.4)	5 (13.5)	30 (81.1)	
	Nurses	13 (6.6)	23 (11.7)	161 (81.7)	
	Pharmacists	32 (86.5)	2 (5.4)	3 (8.1)	
	Nurses	109 (55.4)	27 (13.7)	61 (31)

### Practices related to ADR reporting

**Fig. 5:**
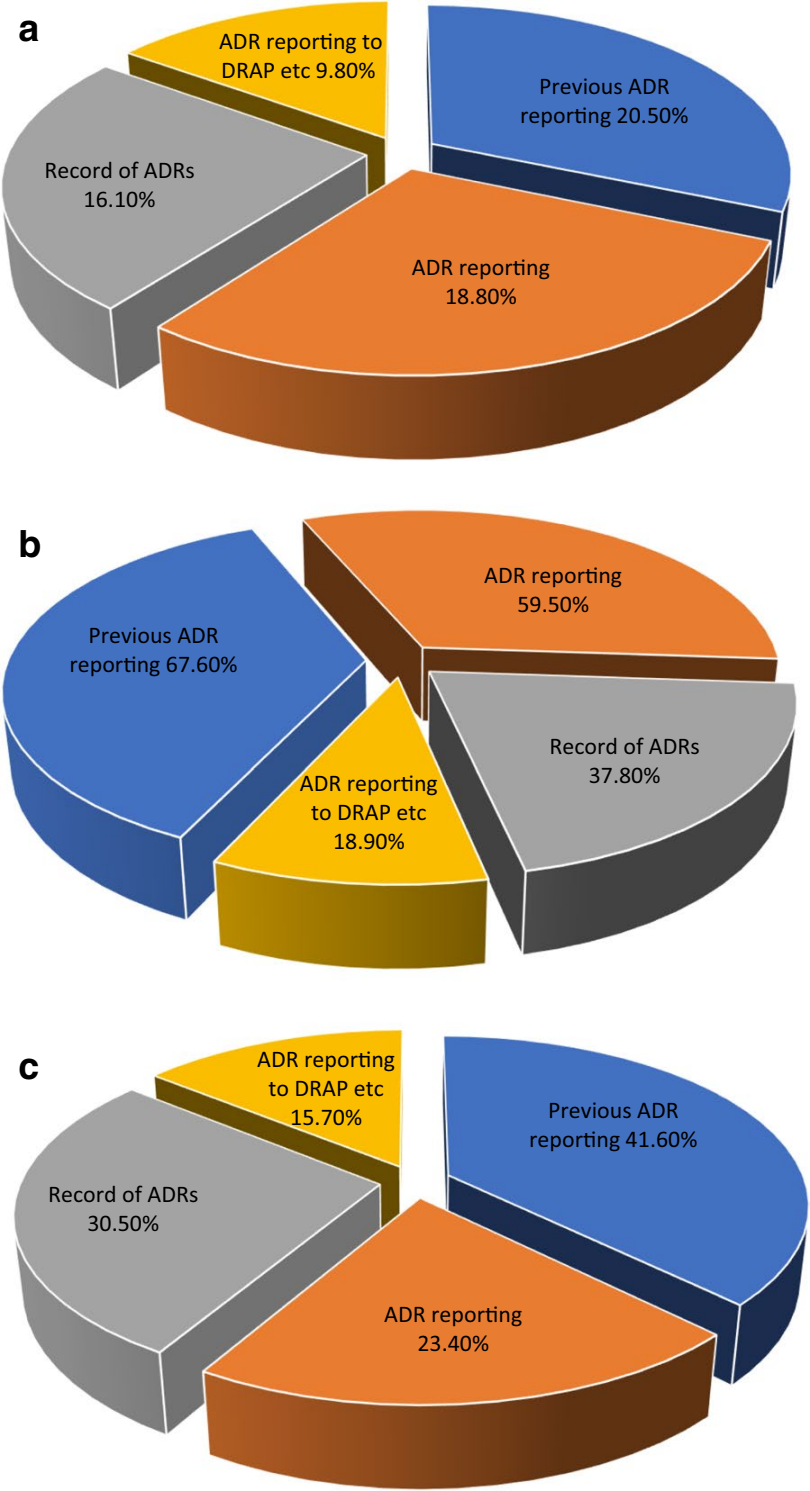
**a** Practices of ADR reporting (by physicians). **b** Practices of ADR reporting (by pharmacists). **c** Practices of ADR reporting (by nurses)

When respondents were asked about their practices regarding ADR reporting in hospitals, 79.5% physicians (*n* = 89, *p* < 0.005) and 58.4% nurses (*n* = 115) stated that they did not report any ADR, while 67.6% of the pharmacists (*n* = 25) stated that they reported ADRs in their workplace. Similarly, only 18.8% physicians (*n* = 21) and 23.4% nurses (*n* = 46) reported ADRs in the last 12 months, contrary to this, 59.5% of pharmacists (*n* = 22) reported ADRs in the last 12 months. About 83.9% physicians (*n* = 94), 62.2% pharmacists (*n* = 23) and 69.5% nurses (*n* = 137) never kept records of ADR. Only 9.8% physicians (*n* = 11), 18.9% pharmacists (*n* = 7) and 15.7% nurses (*n* = 31) sent a suspected ADR reports to the DRAP or to the drug manufacturer, see Table 6 and Fig. 5a–c.

**Table 6:** Practices related to ADR reporting

Practices	Category	Yes	No	*p* value
		Frequency (percentage)	Frequency (percentage)	
		*n* (%)	*n* (%)	
Have you ever reported an ADR before?	Physicians	23 (20.5)	89 (79.5)	0.000
	Pharmacists	25 (67.6)	12 (32.4)	
	Nurses	82 (41.6)	115 (58.4)	
Have you ever reported an ADR in the last 12 months?	Physicians	21 (18.8)	91 (81.2)	0.125
	Pharmacists	22 (59.5)	15 (40.5)	
	Nurses	46 (23.4)	151 (76.6)	
Do you keep records of ADR?	Physicians	18 (16.1)	94 (83.9)	0.004
	Pharmacists	14 (37.8)	23 (62.2)	
	Nurses	60 (30.5)	137 (69.5)	
Have you ever sent a suspected ADR report to DRAP or manufacturer?	Physicians	11 (9.8)	101 (90.2)	0.301
	Pharmacists	7 (18.9)	30 (81.1)	
	Nurses	31 (15.7)	166 (84.3)

### Practices related to the modes of ADR reporting

**Fig. 6:**
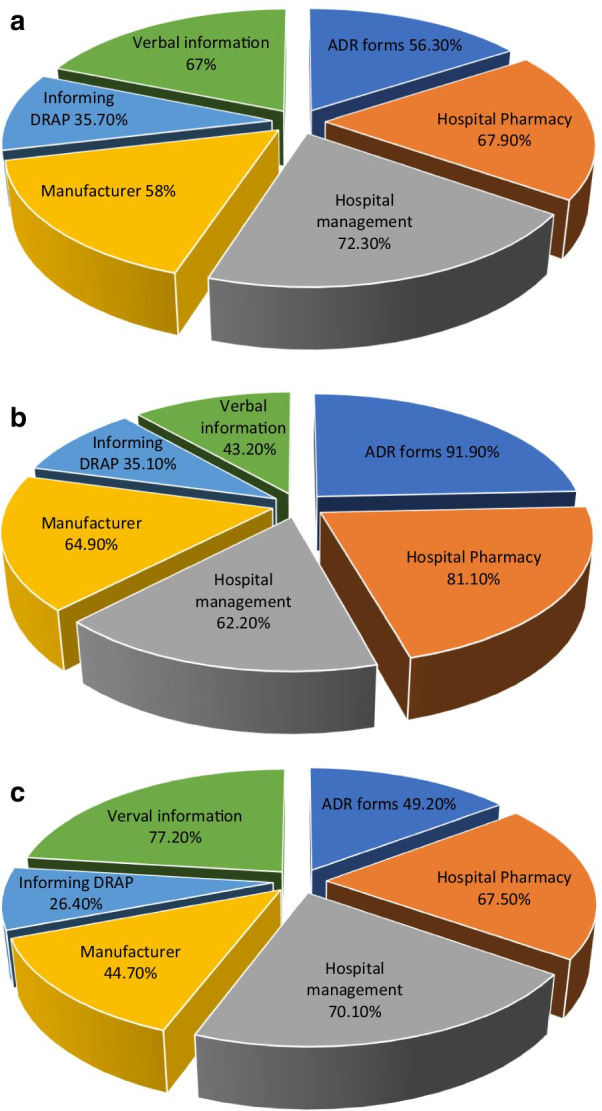
**a** Practices related to the modes of ADR reporting (by physicians). **b** Practices related to the modes of ADR reporting (by pharmacists). **c** Practices related to the modes of ADR reporting (by nurses)

When respondents were asked about the modes of ADR reporting in hospital, 67% physicians (*n* = 75), 77.2% nurses (*n* = 152), reported the ADRs verbally, during the discussions/meetings, while 56.8% pharmacists (*n* = 21) did not verbally report about ADRs. About 56.3% physicians (*n* = 63) and 91.9% pharmacists (*n* = 34) reported an ADR on the ADR reporting forms, while 50.8% nurses (*n* = 100) did not report on ADR forms, available in the hospitals. Among all participants 67.9% physicians (*n* = 76), 81.1% pharmacists (*n* = 30) and 67.5% nurses (*n* = 133) reported to hospital pharmacy about an ADR. Similarly, 72.3% physicians (*n* = 81), 62.2% pharmacists (*n* = 23) and 70.1% nurses (*n* = 138) reported ADRs directly to the hospital management. Moreover, 58% physicians (*n* = 65) and 64.9% pharmacists (*n* = 24) reported ADRs to the relevant manufacturer, while 55.3% nurses (*n* = 109) did not report to the manufacturer. Similar trend was observed in the case of 64.3% physicians (*n* = 72), 64.9% pharmacists (*n* = 24) and 73.6% nurses (*n* = 145) who did not reported ADR through the online portal, as shown in Table 7 and Fig. 6a–c.

**Table 7:** Practices related to the modes of ADR reporting

Mode(s) of ADR reporting	Category	Yes	No
		Frequency (percentage)	Frequency (percentage)
		*n* (%)	*n* (%)
Verbal information	Physicians	75 (67.0)	37 (33.0)
	Pharmacists	16 (43.2)	21 (56.8)
	Nurses	152 (77.2)	45 (22.8)
ADR forms	Physicians	63 (56.3)	49 (43.7)
	Pharmacists	34 (91.9)	3 (8.1)
	Nurses	97 (49.2)	100 (50.8)
Direct reporting to hospital pharmacy	Physicians	76 (67.9)	36 (32.1)
	Pharmacists	30 (81.1)	7 (18.9)
	Nurses	133 (67.5)	65 (32.5)
Direct reporting to hospital management	Physicians	81 (72.3)	31 (27.7)
	Pharmacists	23 (62.2)	14 (37.8)
	Nurses	138 (70.1)	59 (29.9)
Informing the manufacturer	Physicians	65 (58.0)	47 (42)
	Pharmacists	24 (64.9)	13 (35.1)
	Nurses	88 (44.7)	109 (55.3)
Reporting through online forms	Physicians	40 (35.7)	72 (64.3)
	Pharmacists	13 (35.1)	24 (64.9)
	Nurses	52 (26.4)	145 (73.6)

## Discussion

The current study has focused on HCPs including physicians, pharmacists, and nurses, who were working in tertiary care hospitals of Lahore regarding their knowledge, attitude, and practices about pharmacovigilance. The response rate of our survey came out as 90.10%, which is extremely high for a survey response as compared to other studies on the same issue [29, 30, 33–35]. We employed a face-to-face survey, which allows the researcher to have better control over the data collection process and its quality, while telephone, mail or electronic surveys make it difficult to get the data [36]. The proportion of HCPs involved in this study reflected that a major portion of participants was comprised of females and nurses, followed by physicians and pharmacists. This could be supported by the findings from the studies conducted by Bule et al. [37] and Shanko et al. [38], whereby the large number of participants were nurses. Similar to a study by Santosh et al. [39], only females are appointed as a nursing practitioner in public healthcare settings in Pakistan. Regarding age, qualification, and experiences of participants, most of the participants were young, graduates and had at least 4 years of experience [40]. The role of clinical pharmacists in Pakistan is still underutilized and thus the number of appointed clinical pharmacists in tertiary care public hospitals is far less than the recommended number by the WHO. This is the reason, why few pharmacists participated in our study, as in public hospitals many positions are still vacant to be filled [41].

Drugs are the most common treatment interventions and hence requires rational use. If the safety of drug is not considered properly, it may lead to the consequences which might ranges from lifetime disability to the mortality. Likewise, if drug related ADRs are reported, safety of drugs can be improved [42]. In the present study, the majority of the pharmacists showed better knowledge towards ADR reporting as compared to the physicians and nurses. This is similar to studies conducted by Li et al. [41] and Su et al. [43], where pharmacists were able to define ADR more appropriately than physicians and nurses. This is because, during training, pharmacists study medicines and their effects much extensively, while physicians obtain minor knowledge about drugs and pharmacotherapy [5, 44]. The majority of the physicians and pharmacists were more concerned about ADRs, which are serious, including hospitalization, causing death or disability or reactions to newly marketed products, while nurses were even concerned about the reporting of both the minor and major types of reactions to drugs [29]. The similar findings were observed in a study by AlShammari and Almoslem [45], which indicated that 64% of HCPs were only concerned about the reporting of serious ADRs. This perception could be due to the reason that usual or minor ADRs are inevitable and do not cause much harm; however, serious or life-threatening reactions may endanger the life of patient, and thus should be reported.

There are 166 countries, which are either full or partial members of the Programme for International Drug Monitoring (PIDM) by Uppsala Monitoring Centre (UMC). In many member countries, the hospitals have a pharmacovigilance center responsible for the monitoring and recording of ADR. These centers then communicate about ADRs to the national pharmacovigilance center and ultimately to the UMC in Sweden receives the ADR reports. In our study, among all HCPs, only the pharmacists had good knowledge about pharmacovigilance system, recently established under the umbrella of DRAP [46] and were aware of the definition of pharmacovigilance (81.08%), its purpose (91.89%) and national pharmacovigilance center (72.97%), while physicians and nurses did not. It was very similar to the studies from Saudi Arabia, where pharmacists had a high level of awareness than physicians and nurses regarding the pharmacovigilance system [45, 47]. A study by Abdel-Latif and Abdel-Wahab [35] has indicated that only 39.6% of HCPs were aware of the NPC, of which 27.2% were nurses, while 39.2% were physicians and 70.27% were pharmacists. However, these findings disagreed with a study by Suyagh et al. [33], which showed that pharmacists had poor knowledge about pharmacovigilance system. Overall pharmacists in the present study have shown good knowledge about ADR reporting and its system. This could be due to the reason that pharmacovigilance and ADRs are included in the pharmacy curriculum but are partially covered in the curriculum of physicians and nurses [20]. However, the findings suggest that frequent sensitization and trainings should be conducted among all HCPs and drug safety notifications should be disseminated to the healthcare centers.

The attitudes of HCPs are considered pivotal for the reporting of an ADR, thus a positive attitude may encourage the prompt reporting of an ADR. The current study explored the attitudes of all HCPs towards pharmacovigilance activities and showed that overall, all HCPs had a positive attitude towards pharmacovigilance activities in general and ADRs reporting in particular. Although the majority of the participants did not report ADRs in their work setting, they expressed their willingness to report, provided, they are motivated to perform such activities [48]. Among all, most pharmacists (70.2%), and nurses (76.1%) considered ADR reporting as their professional responsibility, similar to a study by Su. et al. [43], where the majority of HCPs considered reporting as professional responsibility [44]. The findings are similar to many studies, where most pharmacists considered ADR reporting as their professional responsibility [43, 44, 49]. Contrary to this, a study from Kuwait has indicated that physicians considered ADR reporting as their responsibility [50]. These findings have indicated that knowledge and awareness about ADR reporting alone cannot serve the purpose and it is mandatory to involve HCPs in ADR reporting on practical grounds.

Many low- and middle-income countries are facing the challenge of low ADR reporting, as low ADR reporting generates minimum signals and thus lacks the pharmacovigilance data [51]. The participants in our study had similar practices for ADR reporting, such as physicians and nurses did not report any ADR in the last 12 months, only 67.6% pharmacists reported ADRs. However, none of each category kept the records of an ADR and few of them ever sent any ADR report to DRAP or drug manufacturer. The practice of not keeping an ADR reporting documented the poor practice among these HCPs, which ultimately could lead to events of drug safety problems [52]. Several studies have shown similar trends in which the majority of HCPs did not report any ADR, although they encountered many during their practice [25, 41, 53, 54].

Participants in our study identified different modes of ADR reporting, including both verbal and non-verbal. It was seen in our survey that the majority of the participants including physicians and nurses have verbally reported ADRs to the concerned person or hospital pharmacy or hospital management, while some physicians and majority pharmacists reported an ADR on the ADR reporting forms. A study conducted in Northern Nigeria has found that healthcare professionals working in a tertiary care setting had similar practices of reporting an ADR [26]. It was also observed that majority pharmacists and physicians reported ADRs either to the manufacturer or DRAP, while nurses did not. This might be due to the fact that nurses had no direct communication with the regulatory authority or manufacturer and this could be due to the perception, that the nurses are not considered as a competent authority to report or communicate an ADR to the manufacturer or regulatory authority [25]. A study from Nepal has revealed that nurses are authorized to report only to seniors and were not encouraged to report independently as seen in the present study [40].

Though all HCPs help in improving ADR reporting, but hospital pharmacists can play a crucial role in this regard, as the majority of ADRs occur in a hospital or could lead to hospitalization [43]. Being a medicines expert, the pharmacist has an important role to ensure the safety of medicines by detecting and reporting ADRs [55]. The role of the pharmacist has evolved worldwide over the past few decades, depending upon the healthcare system, which varies from the dispenser to the custodian of drug safety [5, 55, 56]. Evidence from research suggests that hospital pharmacists not only can detect and report ADRs but help in the prevention of ADRs related fiscal burdens [57, 58]. Furthermore, pharmacists having a clinical background and working in close engagement with prescribers and patients, are in a better position to understand the suspected ADRs [59, 60]. Thus, a clear line of responsibility for each HCP regarding the medicines safety provision could gear the healthcare system towards betterment.

### Limitations

The study was carried out in Lahore; therefore, the results of the present study cannot be generalized to represent the other provinces in the country. However, Punjab is the most developed province and Lahore with a population of over 15 million people (highly populated city of Punjab province) it is the second biggest city in the country. Hence it is expected that the results in the other parts would not be very different.

### Recommendations

The findings from the present study suggest that medicines safety related knowledge and ADR reporting-based training should be provided to all HCPs. To do so, a system of hands-on training and workshops regarding dealing with ADR events must be established at hospital level. Besides, collaboration between academic institutes, drug manufacturers, drug regulatory authority and HCPs should be increased to sensitize ADR reporting related practices.

## Conclusion

The current study has shown that among all HCPs, pharmacists had better knowledge about ADR reporting and pharmacovigilance. All HCPs had a positive attitude and inclination towards reporting an ADR, but the discrepancies were observed in the practices related to ADR reporting. Based on the above, strategies are needed to educate, train and empower the HCPs in the area of pharmacovigilance.

## Data Availability

The datasets used and/or analyzed during the current study are available from the corresponding author on reasonable request.
